# Cardiovascular Autonomic Dysfunction Is the Most Common Cause of Syncope in Paced Patients

**DOI:** 10.3389/fcvm.2019.00154

**Published:** 2019-10-25

**Authors:** Ekrem Yasa, Fabrizio Ricci, Hannes Holm, Torbjörn Persson, Olle Melander, Richard Sutton, Artur Fedorowski, Viktor Hamrefors

**Affiliations:** ^1^Department of Clinical Sciences, Lund University, Malmö, Sweden; ^2^Department of Cardiology, Skåne University Hospital, Malmö, Sweden; ^3^Institute of Cardiology, University “G. d'Annunzio”, Chieti, Italy; ^4^Department of Neuroscience and Imaging, ITAB - Institute Advanced Biomedical Technologies, University “G. d'Annunzio”, Chieti, Italy; ^5^Department of Internal Medicine, Skåne University Hospital, Malmö, Sweden; ^6^National Heart and Lung Institute, Imperial College, Hammersmith Hospital, London, United Kingdom

**Keywords:** pacemaker, pacing, syncope, orthostatic intolerance, cardiovascular autonomic tests

## Abstract

**Introduction:** Syncope and orthostatic intolerance in paced patients constitute a common clinical dilemma. We, thus, aimed to determine the etiology of syncope and/or symptoms of orthostatic intolerance in paced patients.

**Methods:** Among 1,705 patients with unexplained syncope and/or orthostatic intolerance that were investigated by cardiovascular autonomic tests, including Valsalva *maneuver*, active standing, carotid sinus massage, and tilt-testing, 39 patients (2.3%; age 65.6 years; 39% women) had a cardiac implantable electronic device (CIED). We explored past medical history, diagnoses found during cardiovascular autonomic tests, and the further clinical workup, in case of negative initial evaluation.

**Results:** An etiology was identified during cardiovascular autonomic tests in 36 of the 39 patients. Orthostatic hypotension (*n* = 16; 41%) and vasovagal syncope (*n* = 12; 31%) were the most common diagnoses. There were no cases of pacemaker dysfunction. The original pacing indications followed guidelines (sick-sinus-syndrome in 16, atrioventricular block in 16, atrial fibrillation with bradycardia in five). Twenty-two of the 39 patients (56%) had experienced syncope prior to the original *CIED* implantation. Orthostatic hypotension was diagnosed in seven (32%) and vasovagal syncope in nine (41%) of these patients. Of the 17 patients that had not experienced syncope prior to the original CIED implantation, nine patients (53%) were diagnosed with orthostatic hypotension and vasovagal syncope was diagnosed in three (18%). Of the 39 patients, two had implantable cardioverter-defibrillators to treat malignant ventricular arrhythmias diagnosed after syncopal episodes.

**Conclusion:** Cardiovascular autonomic tests reveal the etiology of syncope and/or orthostatic intolerance in the majority of paced patients. The most common diagnosis was orthostatic hypotension (40%) followed by vasovagal syncope (30%), whereas there were no cases of pacemaker dysfunction. Our results emphasize the importance of a complete diagnostic work-up, including cardiovascular autonomic tests, in paced patients that present with syncope and/or orthostatic intolerance.

## Introduction

Syncope is defined as transient loss of consciousness (T-LOC) due to cerebral hypoperfusion, with a rapid onset, short duration, and spontaneous complete recovery (1, 2). For most syncopal events, three main mechanisms may be encountered: reflex syncope, orthostatic hypotension, and cardiac syncope, the latter including bradyarrhythmia as the predominant mechanism (1, 2). Although cardiac pacing is usually very successful in cardiac syncope due to bradyarrhythmia, with syncope recurrence rate of about 5% over 5 years (3, 4), successful pacemaker therapy in reflex syncope of cardioinhibitory type, meaning an asystole longer than 3 s or bradycardia below 40 beats per min, may be challenging (5). In case of concurrent hypotensive tendency, which may be observed as a significant decrease in blood pressure in standing position during head-up tilt test (HUT) (6), the syncope recurrence rate may be as high as 25–50%. In contrast, normal blood pressure response during HUT (tilt-negative) heralds pacing efficacy being almost the same as in primary bradyarrhythmia (5, 6). Thus, cardiac pacing is an effective treatment against syncope when applied in patients with either primary cardiac bradyarrhythmia or in the cardioinhibitory form of reflex syncope, with only a modest hypotensive tendency or so-called “vasodepressor reflex component”.

This approach has been confirmed in the Syncope Unit Project (SUP)-2 reports (7, 8) and current guidelines recommend pacing reflex syncope in selected patients >40 years with recurrent attacks, absence of prodrome and traumatic falls (1). When syncope is unexplained, a stepwise algorithm has been proposed with cardiovascular autonomic assessment as initial stage, and prolonged ECG monitoring by insertable cardiac monitor (ICM) as the next stage, if required (8). However, unexplained syncope and/or orthostatic intolerance in patients with an already implanted pacemaker constitutes a diagnostic and therapeutic challenge and studies addressing clinical management in such patients are sparse. In the current study we, thus, explored the etiology of unexplained recurrent syncope and/or orthostatic intolerance in paced patients.

## Materials and Methods

### Study Setting and Population

The patients in the current study were all from The Syncope Study of Unselected Population in Malmö (SYSTEMA). SYSTEMA was initiated to investigate systematically and manage patients with unexplained syncope (9). Between August 2008 and December 2016, a total of 1,705 patients with suspected syncope i.e., unexplained T-LOC by initial evaluation, who were referred to the tertiary Syncope Unit of Skåne University Hospital, Malmö, Sweden, were enrolled. All 1,705 patients underwent cardiovascular autonomic assessment including carotid sinus massage (CSM), HUT and Valsalva maneuver (1, 2). Along with the main syncope workup, additional tests may have been carried out, including exercise, and external long-term ECG, echocardiography, coronary angiography, brain imaging, and EEG, whenever appropriate. If carotid bruits were detected during admission or hospitalization, a carotid duplex ultrasonography was performed ahead of autonomic tests to rule-out significant carotid artery stenosis.

### Cardiovascular Autonomic Test Examination Protocol

The patients were asked to take their regular medication and fast for 2 h before the test, although they were allowed to drink water without restriction. Prior to examination, the patients were asked to complete a questionnaire, which explored past medical history, duration, frequency and features of syncope-related symptoms, smoking status, and current pharmacological treatment. The cardiovascular autonomic tests included CSM, if appropriate (i.e., if age ≥ 40 years and no contraindications), according to Newcastle protocol (10). In brief, CSM was performed in the supine position using firm longitudinal massage of the right carotid sinus at the site of maximal pulsation 5–10 s while observing symptoms, blood pressure and RR-intervals. If right CSM in the supine position was non-diagnostic (i.e., no asystole > 3 s and no fall in SBP > 50 mmHg), left CSM was performed in the supine position, and then right and left CSM in 70° head-up tilt position.

Head-up tilt-table test was performed at 60–70° including optional nitroglycerin provocation according to the Italian protocol (11). Thus, nitroglycerin (400 μg spray sublingually) was administered first after 20 min of passive HUT if syncope had not occurred and the hemodynamic parameters were stable that is no hypotension (SBP <90 mmHg). Beat-to-beat blood pressure (BP) and electrocardiogram (ECG) were continuously monitored using a non-invasive validated method (Nexfin monitor, BMEYE, The Netherlands), and subsequently analyzed offline using a dedicated program provided by the monitor manufacturer. The Regional Ethical Review Board in Lund, Sweden accepted the study protocol (ref no. 82/2008), and all study participants gave their written informed consent.

### Diagnostic Criteria of Orthostatic Hypotension, Carotid Sinus Syndrome, and Reflex Syncope

The following diagnostic criteria were applied: a) reproduction of symptoms (dizziness, lightheadedness, pre-syncope and syncope), if patients were able to recall conditions preceding syncope, and b) conventional criteria of orthostatic hypotension (OH), carotid sinus syndrome (CSS), and vasovagal reflex syncope (VVS) (1, 2). Briefly, OH was defined as a sustained decrease in systolic BP (SBP) ≥ 20 mmHg and/or decrease in diastolic BP (DBP) ≥ 10 mm Hg, or systolic BP < 90 mmHg, CSS as a fall in SBP ≥50 mmHg and/or asystole >3 s with reproduction of syncope/symptoms, while VVS as a reproduction of syncope associated with a characteristic pattern of pronounced hypotension with or without bradycardia/asystole (1, 2). Moreover, an assessment of initial OH was performed by active standing test if the clinical history was suggestive of this disorder.

### Calculations

Following evaluation in the autonomic laboratory (including Valsalva maneuver, active standing, carotid sinus massage, and tilt-testing), the most likely etiology judged by the investigating physician was compiled for all patients. If no likely diagnosis was established during cardiovascular autonomic testing, additional information was retrieved from the medical records of the patients.

The main characteristics of the study population were presented as mean and standard deviation for continuous variables, and percentages for categorical variables, unless otherwise specified. Continuous variables were compared between groups using Student's *t*-test when normally distributed and with Mann-Whitney U-test if not. Proportions among groups were compared using Pearson chi^2^ test. A *P*-value < 0.05 was considered significant. All calculations were performed using IBM SPSS Statistics software version 25.0 (SPSS Inc., Chicago, IL, USA) and GraphPad Prism version 6.00 (GraphPad Software, La Jolla, CA, USA, www.graphpad.com).

## Results

Of the 1,705 patients that were investigated due to unexplained syncope and/or orthostatic intolerance, 39 (2.3%) already had an implanted pacemaker at the time of the evaluation. The original pacing indications in these patients were sick-sinus-syndrome (SSS) in 16 (41%), atrioventricular block in 16 (41 %) and atrial fibrillation with bradycardia in five (12.8%). Twenty-two of the 39 patients (56%) had experienced syncope prior to the original pacemaker implantation. Two patients (one female and one male, aged 81 and 17 years, respectively) had implantable cardioverter-defibrillators due to malignant ventricular arrhythmias. Both these patients had experienced syncope prior to the implantation. Compared with the rest of the SYSTEMA cohort, the patients with a pre-existing pacemaker were older, more often men and were more likely to have cardiovascular disease (Table 1).

**Table 1:** Patient characteristics (*n* = 1,705) at the time of initial evaluation stratified according to pacemaker status.

	**Patients with pacemakers at the time of evaluation (*n* = 39)**	**Rest of SYSTEMA cohort (*n* = 1,666)**	***P*-value**
Age, years	65.6 (19.9)	51.8 (21.8)	<0.001
Sex, % female	38.5	60.7	0.005
Reported history of			
Syncope, %	84.6	91.5	0.127
Dizziness, *n* %	74.4	72.6	0.811
Number of syncope episodes, md [range]	5 [0–250]	4 [0–1,350]	0.278^a^
Duration of symptoms, years, md [range]	6 [0–48]	3 [0–77]	0.058^a^
SBP, mmHg	132.8 (18.7)	131.4 (22.5)	0.71
DBP, mmHg	68.8 (9.1)	71.6 (10.2)	0.091
Resting heart rate, bpm	67.2 (8.1)	70.3 (12.6)	0.028
Hypertension, %	51.3	28.5	0.002
CAD, %	30.8	6.4	<0.001
Atrial fibrillation, %	33.3	6.6	<0.001
Heart failure, %	25.6	3.3	<0.001

a *P-value for Mann-Whitney U-test. Continuous variables were compared between groups using Student's t-test and dichotomous variables were compared according to group using Pearson chi^2^ test, if not otherwise indicated. md, median; SBP, systolic blood pressure; DBP, diastolic blood pressure; CAD, coronary artery ldisease*.

**Figure 1:**
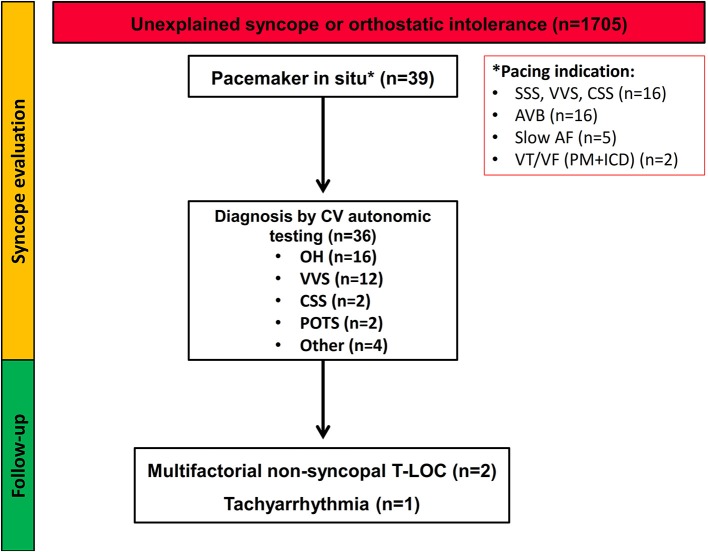
Flow-chart of the study population. The diagram summarizes the diagnostic work-up and follow-up of patients presenting with unexplained syncope or symptoms of orthostatic intolerance. SSS, sick sinus syndrome; VVS, vasovagal syncope; CSS, carotid sinus syndrome; POTS, postural orthostatic tachycardia syndrome; AVB, atrioventricular block; AF, atrial fibrillation; VT, ventricular tachycardia; VF, ventricular fibrillation; PM, pacemaker; ICD, implantable cardioverter defibrillator; T-LOC, transient loss of consciousness.

Following evaluation in the autonomic laboratory (including Valsalva maneuver, active standing, carotid sinus massage, and tilt-testing), an etiology was identified in 36 of the 39 patients, of which OH was the predominant diagnosis (Figure 1). Regarding the three patients in whom no etiology could be identified during tilt, further work-up demonstrated ventricular tachyarrhythmia in one; in another, vertigo, dementia and neurodegenerative changes were found and in the third, balance/gait disorder without haemodynamic basis, was considered causative.

Among the 22 patients that had experienced syncope prior to the original device implantation, orthostatic hypotension was diagnosed in seven (32%) and vasovagal syncope in nine (41%) patients. Of the 17 patients that had not experienced syncope prior to the original pacemaker implantation, nine patients (53%) was diagnosed with orthostatic hypotension whereas vasovagal syncope was diagnosed in three (18%). Statistical power calculations indicated insufficient power to detect any statistically significant differences in diagnoses between the 22 patients with prior syncope and the 17 patients without prior syncope.

All patients underwent pacemaker interrogation as an initial part of their assessment. There were no cases of pacemaker dysfunction. No paced patient received an ICM for diagnosis.

Most patients (28/39) were aged 60 years or more. In these patients, orthostatic hypotension was diagnosed in 50%, whereas vasovagal syncope was dominant in patients under 60 years of age. Cardiovascular autonomic tests indicated the etiology in all patients under 60 years of age. Results stratified according to age over/under 60–years appear in Tables S1–S3.

## Discussion

In the current study we have shown that:

- A likely etiology of syncope and/or orthostatic intolerance in patients with pacemakers can be successfully identified by cardiovascular autonomic tests, including head-up-tilt, carotid sinus massage and Valsalva maneuver.
- The most common etiologies in the unexplained group are orthostatic hypotension (preferentially in older subjects) and vasovagal syncope (preferentially in younger subjects). There were no cases of pacemaker dysfunction in our cohort.

The pacing literature has focused on symptoms and ECG diagnosis in order to select patients for successful pacing therapy. Recurrent syncope or orthostatic intolerance in paced patients has had less attention. Early series raised the possibility of autonomic causes, although a full range of autonomic investigations was not available to those investigators (12, 13). Using a prospective investigational protocol including cardiovascular autonomic tests, we have been able to provide insights into analysis of the etiology of recurrent syncope and/or orthostatic intolerance in paced patients. Orthostatic hypotension or vasovagal syncope was the etiology in seven of ten patients. Notably, orthostatic hypotension was more common among paced patients (41%) than in the rest of the SYSTEMA cohort (27%) and the proportion of patients in whom no cause could be identified during tilt was lower (8% compared with 22%). Regarding the finding of vasovagal syncope, sick sinus syndrome was a common original pacing indication (41%), thus, it should be considered that many of these paced patients show the “extrinsic” form (13), implying a reflex mechanism for syncope with a vasopressor component (1). Importantly, in paced patients with cardioinhibitory vasovagal syncope, the anti-bradycardia stimulation cannot treat the vasodepressor component, which was undetected, even on tilt if performed before implantation, by the severe bradycardia/asystole. Performance of tilt prior to pacing must now be considered as a risk of syncope recurrence tool, if positive, recurrence of syncope is substantially more likely (6). While the initial pacing indications followed guidelines in all patients, pacing offers little or no help for the vasodepressor component of vasovagal syncope and in orthostatic hypotension, thus constitutes the basis of recurrent syncope.

Of note, assessment of pacing function (performed in all patients) revealed no cases of dysfunction. Rather, our study affirms the importance of a comprehensive diagnostic work-up according to recent syncope guidelines (1, 2) also in patients with pre-existing pacemakers that present with recurrent syncope and/or orthostatic intolerance. Interestingly, cardiovascular autonomic tests indicated the etiology in all eleven patients under 60 years of age, suggesting that cardiovascular autonomic test may be particularly valuable in this age group. Concentrating expertise in a dedicated facility (“Syncope Unit”) (1) offers increased diagnostic and therapeutic efficacy, as cardiovascular autonomic tests are not widely available and cardiologists may have limited knowledge of test interpretation.

In this study, we did not use Closed Loop pacing as was done in the SPAIN trial (14). This pacemaker senses right ventricular volume indirectly by measuring its impedance. When impedance increases by decrease in right ventricular volume, as occurs in vasovagal syncope due to diminishing cardiac output and venous return, pacing is triggered. This detected change precedes bradycardia/asystole in almost all vasovagal syncope, thus, the trigger for pacing is earlier in the reflex than waiting for later occurring bradycardia. The favorable results of the SPAIN trial suggest that this means of triggering pacing may offer more benefit. The BIOSYNC study, a randomized controlled trial of CLS vs. standard DDD pacing has almost completed recruitment (15).

We acknowledge some study limitations. Firstly, this is a single-center observational study with limited sample size, requiring our results to be confirmed. Secondly, our study is of a selected group referred to a tertiary syncope unit, thus, it may not reflect the etiology of a wider syncope population. The relatively low proportion of patients with an existing pacemaker at the time of entry into the cohort (2.3%) may be explained by the fact that only subjects with unexplained syncope and/or orthostatic had been referred to the syncope unit. Thus, the SYSTEMA population is a selected group in whom syncope etiology could not readily be determined and/or the patient adequately managed by the referring physician. Thirdly, our examination protocol did not include additional autonomic tests such as the Valsalva maneuver or baroreceptor sensitivity test in all patients.

## Conclusion

In conclusion, we have shown that cardiovascular autonomic tests indicate the etiology of syncope and/or orthostatic intolerance in the majority of paced patients. The most common diagnosis is orthostatic hypotension (40%) followed by vasovagal syncope (30%), which emphasizes the importance of a full diagnostic work-up in paced patients that present with recurrent syncope and/or orthostatic intolerance.

## Data Availability Statement

The datasets generated for this study are available on request to the corresponding author.

## Ethics Statement

The studies involving human participants were reviewed and approved by The Regional Ethical Review Board in Lund, Sweden (ref no. 82/2008). The patients/participants provided their written informed consent to participate in this study.

## Author Contributions

EY, RS, AF, and VH: concept and design. EY, FR, HH, TP, OM, RS, AF, and VH: data analysis, interpretation, drafting article, critical revision of article, and approval of article. AF and VH: statistics. OM, AF, and VH: funding secure. EY, TP, and AF: data collection. FR, HH, and TP: other.

### Conflict of Interest

AF reports personal fees from Cardiome Corp. AF and OM report patent royalties from ThermoFisher outside the submitted work. VH reports educational congress grant from Boston Scientific Inc. RS reports personal fees and other from Medtronic Inc., St. Jude Medical Inc. (Abbott Laboratories) outside the submitted work. RS performs consultancy for Medtronic Inc. RS is a member of the speaker's Bureau St. Jude Medical/Abbott Inc., RS is shareholder in Boston Scientific Inc., Edwards Lifesciences Inc., and AstraZeneca PLC.

The remaining authors declare that the research was conducted in the absence of any commercial or financial relationships that could be construed as a potential conflict of interest.
