# Supplementary material for: Cardiovascular Autonomic Dysfunction Is the Most Common Cause of Syncope in Paced Patients
Source: Front Cardiovasc Med. 2019 Oct 25;6:154. doi: 10.3389/fcvm.2019.00154 (PMC6823818; doi:10.3389/fcvm.2019.00154)
Supplement: Supplementary file 1 [file Data_Sheet_1.docx]

**Supplementary tables**

Table S1: The clinical characteristics of patients with a pacemaker at the time of syncope evaluation

|  | All (n=39) | Age ≤ 60 years (n=11) | Age > 60 years (n=28) |
| --- | --- | --- | --- |
| Age, years | 65.6 (19.9) | 39.3 (16.4) | 75.9 (8.0) |
| Sex, % female | 38.5 | 54.5 | 32.1 |
|  |  |  |  |
| Reported history |  |  |  |
| Syncope, % | 84.6 | 90.9 | 82.1 |
| Dizziness, n % | 74.4 | 72.7 | 75.0 |
| Number of syncope episodes, md [range] | 5 [0-250] | 20 [0-100] | 5 [0-250] |
| Duration of symptoms, md [range] | 6 [0-48] | 10 [1-48] | 5 [0-41] |
|  |  |  |  |
| SBP, mmHg | 132.8 (18.7) | 124.4 (18.8) | 136.1 (17.9) |
| DBP, mmHg | 68.8 (9.1) | 70.4 (8.8) | 68.2 (9.3) |
| Resting heart rate | 67.2 (8.1) | 68.8 (10.9) | 66.6 (6.8) |
|  |  |  |  |
| Hypertension | 51.3 | 18.2 | 64.3 |
| CAD | 30.8 | 18.2 | 35.7 |
| Atrial fibrillation | 33.3 | 0 | 46.4 |
| Heart failure | 25.6 | 18.2 | 28.6 |
| Displayed as mean (standard deviation) unless otherwise specified.  md = median; SBP = systolic blood pressure; DBP = diastolic blood pressure; CAD = coronary artery disease | | | |
|  |  |  |  |

Table S2: Original pacing indications in patients with a pacemaker at the time of syncope evaluation

|  | All  (n=39) | Age ≤ 60 years  (n=11) | Age > 60 years (n=28) |
| --- | --- | --- | --- |
| Sick sinus syndrome, n (%) | 16 (41.0) | 4 (36.4) | 12 (42.9) |
| Atrioventricular block, n (%) | 16 (41.0) | 6 (54.5) | 10 (35.7) |
| AF with bradycardia, n (%) | 5 (12.8) | 0 | 5 (17.9) |
| VT or VF, n (%) | 2 (5.1) | 1 (9.1) | 1 (3.6) |
| AF = atrial fibrillation; VT = ventricular tachycardia; VF = ventricular fibrillation | | | |
|  |  |  |  |

Table S3: Syncope diagnosis in patients with a pacemaker following head up tilt test with carotid sinus massage

|  | All  (n=39) | Age ≤ 60 years  (n=11) | Age > 60 years (n=28) |
| --- | --- | --- | --- |
|  |  |  |  |
| **Diagnosis during HUT + CSM, n (%)** | **36 (92.3)** |  |  |
| Orthostatic hypotension, n (%) | 16 (41.0) | 2 (18.1) | 14 (50.0) |
| Vasovagal syncope, n (%) | 12 (30.8) | 6 (54.4) | 6 (21.4) |
| Carotid sinus syndrome, n (%) | 2 (5.1) | 0 | 2 (7.1) |
| POTS, n (%) | 2 (5.1) | 2 (18.2) | 0 |
| Other, n (%)* | 4 (10.3) | 2 (18.2) | 2 (7.1) |
|  |  |  |  |
| **Diagnosis during follow-up, n (%)** | **3 (7.7)** | **0** | **3 (10.7)** |
| Multifactorial non-syncopal TLOC^#^, n (%) | 2 (5.1) | 0 | 2 (7.1) |
| Tachyarrhythmia | 1 (2.6) | 0 | 1 (3.6) |
|  |  |  |  |
| HUT=head-up tilt; CSM=carotid sinus massage; POTS=postural orthostatic tachycardia syndrome; TLOC=transient loss of consciousness.  * Including initial orthostatic hypotension (n=2), situational syncope (n=1) and general hypotension (n=1) | | | |
|  |  |  |  |
